# Midfrontal theta oscillation encodes haptic delay

**DOI:** 10.1038/s41598-021-95631-1

**Published:** 2021-08-23

**Authors:** Haneen Alsuradi, Wanjoo Park, Mohamad Eid

**Affiliations:** 1 Tandon School of Engineering, New York University, New York City, NY 11201 USA; 2 Engineering Division, New York University Abu Dhabi, Saadiyat Island, Abu Dhabi, 129188 United Arab Emirates

**Keywords:** Neuroscience, Engineering

## Abstract

Haptic technologies aim to simulate tactile or kinesthetic interactions with a physical or virtual environment in order to enhance user experience and/or performance. However, due to stringent communication and computational needs, the user experience is influenced by delayed haptic feedback. While delayed feedback is well understood in the visual and auditory modalities, little research has systematically examined the neural correlates associated with delayed haptic feedback. In this paper, we used electroencephalography (EEG) to study sensory and cognitive neural correlates caused by haptic delay during passive and active tasks performed using a haptic device and a computer screen. Results revealed that theta power oscillation was significantly higher at the midfrontal cortex under the presence of haptic delay. Sensory correlates represented by beta rebound were found to be similar in the passive task and different in the active task under the delayed and synchronous conditions. Additionally, the event related potential (ERP) P200 component is modulated under the haptic delay condition during the passive task. The P200 amplitude significantly reduced in the last 20% of trials during the passive task and in the absence of haptic delay. Results suggest that haptic delay could be associated with increased cognitive control processes including multi-sensory divided attention followed by conflict detection and resolution with an earlier detection during the active task. Additionally, haptic delay tends to generate greater perceptual attention that does not significantly decay across trials during the passive task.

## Introduction

Many systems that involve human–machine interaction, such as teleoperation or virtual reality, are incorporating haptic information to enhance perception and manipulation of the environment^1^. One perceptual attribute that provides an essential basis for haptic-visual integration is haptic delay due to stringent computational and communication needs associated with haptic data^2^. Haptic delay can be defined as the temporal difference between the actual haptic feedback and the expected one. Delayed haptic feedback can seriously disrupt many aspects of the interaction, such as the completion time of manipulation tasks^3^, quality of teleoperation^4^, and the perception of physical properties such as stiffness and friction^5^.

The perceptual consequences of haptic delay have received significant attention through psychophysical studies. Detection thresholds for haptic delays may vary substantially, between 20 and 200 ms, based on the application and the type of haptic interaction (force feedback versus tactile, discrete versus continuous force feedback, and active versus passive interaction)^6^. A haptic-visual study showed that a discrete haptic feedback is noticed as delayed if the delay exceeded 110 ms^6^. In a collaborative virtual environment where haptic information is bi-directionally communicated, haptic feedback delay could be perceived starting from around 50 ms^7^. It is also reported that humans do not perceive delays below 30 ms during continuous haptic interaction^8^. Understanding the experience of perceiving haptic delay is an essential consideration for haptic technology designers to optimize the realism of the haptic experience.

Neurohaptics is an emerging field that strives to understand the complex neural representations provoked in response to touch stimulation^9^. Neural imaging techniques such as fMRI and EEG offer the potential to examine brain activities associated with haptic delay to provide objective, real-time assessment of the haptic delay^9,10^. Compared to other neural imaging techniques such as fMRI, EEG is preferable due to the compatibility with electric devices, relatively low cost, and the ability to measure brain responses with high temporal resolution^9^. Previous studies utilized EEG to examine brain correlates associated with unexpected or mismatched visual stimulation. For instance, an event-related potential (ERP) study found that a negative potential around 200 ms (N200) is pronounced after seeing a visual stimulus that was not expected^11^. Furthermore, in an object selection task in a virtual environment, it was found that the prediction error negativity component of the ERP signal was more pronounced when the user’s hand had unrealistic representation^12^.

Converging EEG studies have unraveled the functional roles that the central and midfrontal areas, and in particular the theta frequency band, play in conflict analysis at the stimulus/response level^13,14^, at the semantic/cognitive level^15,16^ and during cross-modal prediction error processing^17^. An early study found that theta oscillation at the midfrontal region is closely related to cognitive control processes that are needed to evaluate the stream of information from perceived stimuli and prepare the brain’s response accordingly^18^. These processes include multisensory divided attention^19^, conflict detection and resolution and selective suppression^20^. In multimodal interaction such as audiovisual, increased central theta power was observed following incongruent audiovisual stimuli compared with congruent audiovisual stimuli^21^.

Studies on the haptic modality are scarce. A few studies reported that theta synchronization at the midfrontal cortex reflects conflict monitoring and resolution to visual stimuli involving a motor response^20,22^ or an initiated motor movement^23^. Theta oscillation was reported to be more pronounced under incongruent cross-modal stimulation as compared to congruent stimulation under visuotactile matching paradigm using Braille stimulator and a computer screen^24^. In a similar study, visuotactile congruency was tested using two LEDs (visual stimuli) and two vibrotactile motors (tactile stimuli) placed on the thumb and the forefinger; incongruent stimulation induced a significantly greater theta band activity during 300–500 ms after stimulation^25^.

This paper focuses on examining neural correlates associated with delayed force feedback during active and passive tasks. A delay value of 220 ms has been selected for this experiment based on the previously mentioned literature^6,26^; an easily recognizable delay is needed to clearly identify the neural correlates. This decision is complimented by a pilot study that was conducted to make sure the delay is clearly perceivable by the majority of participants (beyond 90% recognition accuracy). ERP and event-related spectral perturbation (ERSP) in the theta band are examined in the central and midfrontal areas, respectively. We also study the impact of delay on the sensory correlates at the contralateral sensorimotor cortex. Three hypotheses are examined: (1) haptic delay elicits theta oscillation that is related to multi-sensory divided attention and conflict-resolution processes, (2) haptic delay has no impact on the sensory correlates, represented by beta rebound, and (3) a learning effect due to the repetitive exposure to the haptic stimulus could be observed through ERP components related to attentional resources devoted to the haptic feedback.

## Methods

### Participants

Nineteen subjects participated in the experiment (ten females, nine males), where 17 of them are aged between 18 and 25 years, one is aged between 25 and 30 years, and one is aged between 30 and 40 years. All participants were right-handed and performed the experiment with their right hand. Also, participants had either a normal vision or corrected-to-normal vision. Around 74% of the participants did not use a haptic device before. Each participant received a compensation voucher worth 30 dollars (USD) for participation. The sample size of the study was not calculated priorly through statistical power analysis. Instead, sample size selection was based on previous EEG studies that investigated sensorimotor processes (Savoie et al.^27^, N = 15; Tan et al.^28^, N = 17; Torrecillos et al.^29^, N = 15; Perfetti et al.^30^, N = 17; Lin et al.^31^, N = 19). The study was carried out with an approved protocol by New York University Abu Dhabi Institutional Review Board (IRB: #HRPP-2019-120) and in accordance with the Declaration of Helsinki, following its guidelines and regulations. Written informed consent was obtained from all participants in accordance with the IRB ethics before enrolling in this study.

### Experimental setup and task

**Figure 1:**
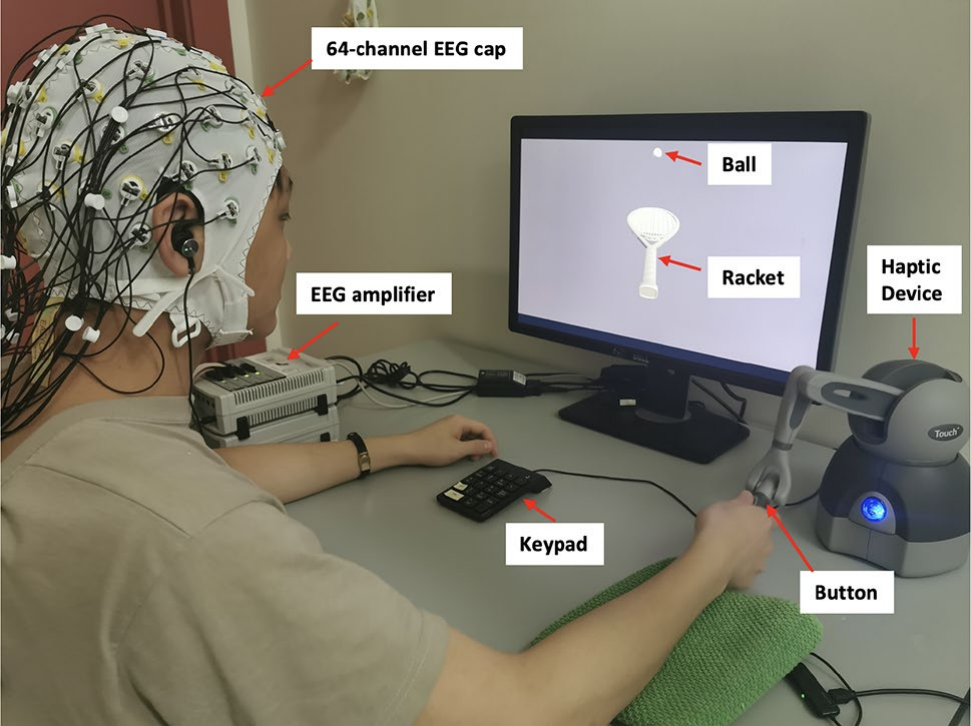
The participant is seated in the experimental room, and the EEG cap is placed on the scalp. Electrodes are connected to the EEG amplifier via a multi-wire planar cable. The participant holds the stylus of the haptic device with their right hand placing their thumb on the button integrated into the stylus (for the passive task). The game is displayed on a computer screen one meter away from the participant. A keypad is placed next to the participant’s left hand to answer if there was a delay or not in the previous trial whenever asked. A folded towel is placed underneath the participant’s arm for better comfortability. The participant is trained on using the haptic device with minimal body movement to avoid excessive EEG artifacts. The participant is asked to stay seated and use one hand to move the wrist up and down (during the active task) while the elbow is rested without movement.

Participants were seated on a chair around one meter in front of a computer screen and were asked to hold the stylus of a haptic device (Geomagic Touch, 3D systems, United States) with their right hand. Participants were told they would participate in a haptic-visual task in which the goal is to bounce a tennis ball using a racket that is controlled by the haptic device. The game was developed using Unity game engine version 2018.4.5f1 (Unity technologies, United States) and Openhaptics Unity toolkit (3D Systems, United States). Participants had to perform passive and active tasks under synchronous visual and haptic stimulation or asynchronous stimulation such that the haptic feedback is delayed by 220 ms. During the active task, participants moved the racket up to bounce the ball, which is initially held stationary above the racket. However, during the passive task, the racket is held passively, and a thumb button-press on the haptic device initiated a free-fall of the tennis ball to collide with the racket. Force feedback was felt upon the ball collision with the racket. The haptic collision was either provided synchronously with the visual collision or 220 ms delayed with respect to the visual collision. Participants were trained on using the haptic device with minimal body movement to avoid excessive motor EEG artifacts. During the active task, participants were asked to move their wrists up or down while the elbow and forearm are rested without movement. Figure 1 shows one of the participants correctly holding the stylus of the haptic device along with the experimental setup.

**Figure 2:**
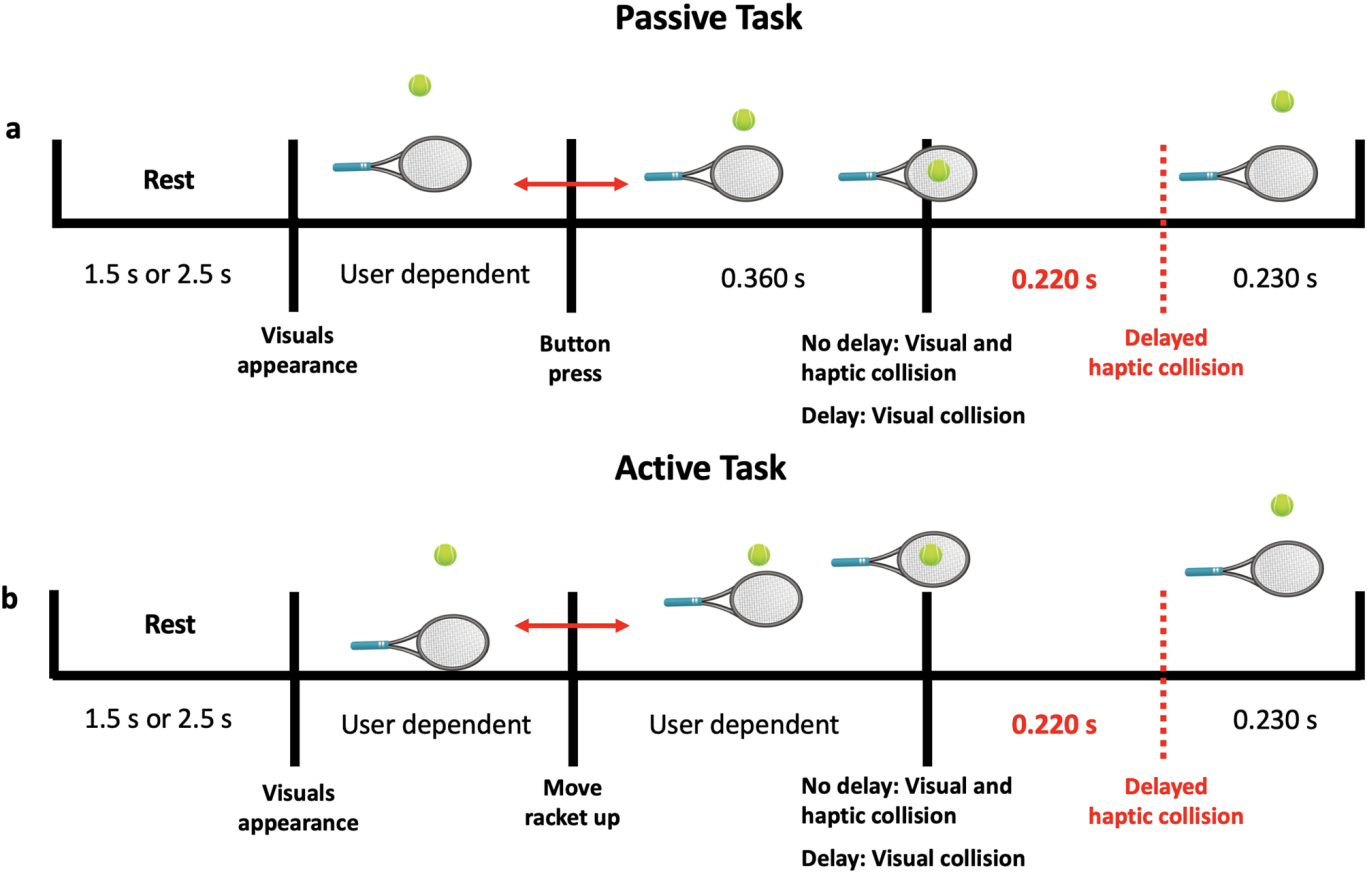
Schematic representation of the experimental task and the sequence of events. Haptic collision is either delayed by 220 ms or synchronous to the visual collision between the racket and the ball. Trials are divided as (50 trials are passive and synchronous, 50 trials are passive with haptic delay, 50 trials are active and synchronous, and 50 trials are active with haptic delay). (**a**) The passive task is initiated with a button press on the stylus of the haptic device. The ball will fall to collide with the racket (**b**) The active task is initiated with an actual movement of the stylus towards the ball. The time taken for the racket to hit the ball depends on the user’s motion.

A single trial consisted of either a 1.5 or 2.5 s rest period (randomized) during which a blank screen was presented, followed by a single bouncing move; Fig. 2 shows the sequence of events in a single trial for both the passive and the active tasks. The experimental session consisted of ten runs, each of which had 20 trials. Five runs were conducted under the passive mode, while the other five runs were conducted under the active mode. Ten trials had delayed haptic feedback within a single run while the other ten had synchronous haptic feedback; trials were sequenced randomly. Thus, in total, each participant performed 200 trials equally divided between the four experimental conditions (passive/active, with/without haptic delay). The experimental runs’ order was counterbalanced across participants to avoid the order effect between the passive and active tasks. The physics of the experiment, including the ball’s weight (60 g) and its bounciness, were kept as usual and expected as possible. This is to provide a realistic haptic experience to the participant. The amplitude and duration of the force feedback were held to constant values, 0.6 N and 60 ms, respectively, in all trials to provide a fair comparison between the different conditions. To make sure participants are well-engaged and to have a general subjective measure of their performance in recognizing the synchronous from asynchronous trials, they were asked to answer if the haptic feedback was delayed or not in 30 trials out of 200 using a keypad. Participants were asked to fill a post-experiment questionnaire to capture their perceptual experience.

### EEG data acquisition

EEG data were recorded at a 1 kHz sampling rate using an EEG amplifier (BrainAmps Standard, Brain Products, Germany) and the Brain Vision Recorder software (BVR; Version 1.21.0201 Brain Products, Germany). The EEG cap had a 64 Ag/AgCL based electrode set (actiCAP snap, Brain Products, Germany), that was placed according to the 10–20 international system such that Cz electrode is placed over the vertex of the participant’s head. The online reference electrode was positioned at the FCz location, while the ground electrode was positioned at the FPz location. Input impedance on each electrode was kept below 15 k$\Omega$ to ensure high-quality signal recordings.

### EEG data pre-processing

The EEG data were pre-processed and analyzed offline using MATLAB release 2019b (MathWorks, United States) and EEGLAB toolbox (v14.1.2)^32^. Four channels that are located at the sides of the head (FT9, FT10, TP9, and TP10) were excluded from the analysis. The data were first filtered between 0.1 and 35 Hz using a zero-phase Hamming windowed sinc FIR filter. The artifact subspace reconstruction (ASR)^33^ method was applied to remove high-amplitude artifacts, including eye blinks, muscle bursts, and physical movement, as well as to reject bad channels and correct them by channel interpolation. Channels were then re-referenced using the Common Average Referencing (CAR) method^34^ while restoring the online reference channel, FCz, to the dataset.

Due to the asynchronous nature of the experimental task and since the initiation of the task is participant-dependent (pressing the button or moving the racket), the EEG data were double epoched. The first epoching was time-locked to the event of visuals appearance on the screen. The time between the appearance of visuals and the visual collision ($\Delta$) was calculated for all trials, and its distribution was plotted. Trials that have $\Delta$ more than four standard deviations away from the mean were discarded (passive: $\Delta = 1105 + 4\times (744)$ ms; active: $\Delta = 1139 + 4\times (928)$ ms); this is to maintain a uniform behavioral trend in the considered trials. The data were then epoched from $-1$ to 6 s around the visuals’ appearance event. The second epoching is performed time-locked to the haptic feedback event, which is the event of interest, with an epoch period between $-1$ and 2 s around the onset of the haptic feedback. Since the rest period is not exactly prior or close to the collision event, the rest period was not used for baseline correction (ERP) or baseline normalization (ERSPs). Additionally, and since we introduce a delay in the haptic feedback in half of the trials, the period preceding the haptic feedback cannot be used as a baseline either. This choice will lead to inconsistent baselines across conditions. Instead, the baseline was selected to be the period that exactly precedes the visual collision event. This choice will guarantee that the baseline is close enough to the onset (haptic collision) while being consistent across all conditions (the state of mind before the collision occurrence). Thus, the data were baseline corrected to the average potential recorded during the 100 ms preceding the visual collision onset (ERP) and baseline-normalized with respect to the 200 ms data that preceded the visual collision onset (ERSPs). Lastly, the infomax algorithm was applied to implement independent component analysis (ICA)^35^, a blind source separation technique that separates signal components by maximizing the statistical independence between them. A component was marked as artifactual if: (1) the time-course of the component showed bogus bursts of activity, and (2) the power spectrum of the component was found to increase with frequency; EEG spectral power is expected to decrease with frequency^36^ as opposed to muscle artifacts’ spectral power which increases with frequency^37^, or (3) the topography of the component was localized at the far edges of the scalp as these could either be eye or muscle artifacts. The clean components were then reflected back to the channel space.

### Electrodes, bands and time-windows selection

For the EEG data analysis, the selection procedure of electrodes, frequency bands, and time windows are based on available literature. As per our hypothesis (1) stated earlier in the introduction, theta power at the midfrontal area plays an essential functional role in multisensory attention and conflict processing^19,20^. Therefore, EEG data were examined at the midfrontal region of interest (ROI) comprising the following electrodes: AFz, F1, Fz, F2, FC1, FCz, FC2. This selection is based on previous investigations of the midfrontal theta oscillations, where chosen electrodes are usually centered around Fz electrode^19,20^. Within the same midfrontal ROI, the ERP component of P200 is reported to be modulated in response to delayed auditory feedback^38^, visuo-haptic mismatch in virtual reality^39^ and in conflict resolution studies^20^. We report the P200 result for FCz electrode, partly because it was the electrode that showed the maximum P200 modulation with the introduction of haptic delay and partly because the previously mentioned EEG studies on delayed feedback and conflict processing focus mainly on this electrode. It was decided to consider the time window between 140 and 280 ms from the haptic feedback onset since it is reported that the P200 peak is observed within this time window^40–42^.

In addition to the midfrontal ROI, EEG data were examined at the contralateral sensorimotor ROI to observe sensory correlates, and the impact of haptic delay on them compared to the cognitive correlates at the midfrontal ROI. Particularly, sensorimotor tasks invoke a drop in beta power during the movement of the effector followed by a post-movement beta rebound^43^ observed over the sensorimotor area. We selected FC1, C1, C3, CP3 electrodes as they overlie the sensorimotor area and are known to best capture the post-movement beta rebound^44,45^.

### EEG data analysis

For ERP analysis, epochs of the same condition were averaged out across all subjects. For time-frequency analysis, epoched EEG data were first spatially filtered; current source density (CSD) transformation was calculated using the surface Laplacian transform (m-constant: 4, head radius: 10 cm, smoothing constant: 10$^{-5}$) implemented as part of the CSD toolbox in Matlab (version 1.1)^46^. There is strong evidence that applying CSD transformation to the EEG activity improves the spatio-temporal features and resolution of the EEG dynamics. Additionally, applying CSD following ICA-based artifact rejection has been shown effective in eliminating undesired muscle-related, and electromyographic activity^47,48^. The second step was to apply Morlet Wavelet transformation^49^ with a cycles range logarithmically increasing from 4 to 20 to maintain better frequency resolution at higher frequencies^50^. The power was then calculated for all frequencies, timepoints, and channels by squaring the amplitude of the transformed signals. The mean was used as a central tendency measure to represent the ERSP data of each participant. The power time series was then normalized through decibel conversion. Finally, the power of the brainwaves in the frequency bands of interest was extracted: theta (4–9 Hz) and beta (13–30 Hz).

### Statistical analyses

The non-parametric randomization test was used for the statistical analysis of EEG data. Randomization testing is a type of surrogate test widely used on EEG signals^51^; they are ideal for null hypothesis testing because they make no assumption on the data distribution (i.e., whether it follows a normal distribution or not). Additionally, randomization tests are relatively more general when compared to the traditional parametric statistical tests as they do not force a specific test statistic on which the statistical inference is based upon (i.e., t- or F-statistic)^52^. All the statistical tests across the manuscript are two-tailed, and the threshold of significance is set to 0.05. Statistical analysis on ERP data was computed by splitting the time window of interest to an equal number of intervals of 20 ms and performing the statistical test on each of the segments separately. For the ERSP data, on the other hand, no predefined time window was decided. Thus, the time between 0 and 600 ms and 0–800 ms was split into 30 and 40 equal intervals of 20 ms for the theta and beta band activations, respectively. Statistical analysis was conducted on each of the time bins to determine if there is a statistical significance between the delayed and synchronous conditions. For both ERP and ERSP, the statistic is computed at the group level such that data are averaged over all trials within the subject.

To limit the inflation of type 1 error due to multiple comparisons in the statistical analysis of both ERP and ERSP data, *p* values were corrected using the false discovery rate (FDR) method^53^. The number of comparisons, k, is equal to the number of time bins in each statistical test (ERP: k = 7; ERSP(theta band): k = 30 and ERSP(beta band): k = 40). For all analyses, we report the FDR corrected *p* values along with the time range during which the statistical significance was found.

## Results

### Sensory correlates

**Figure 3:**
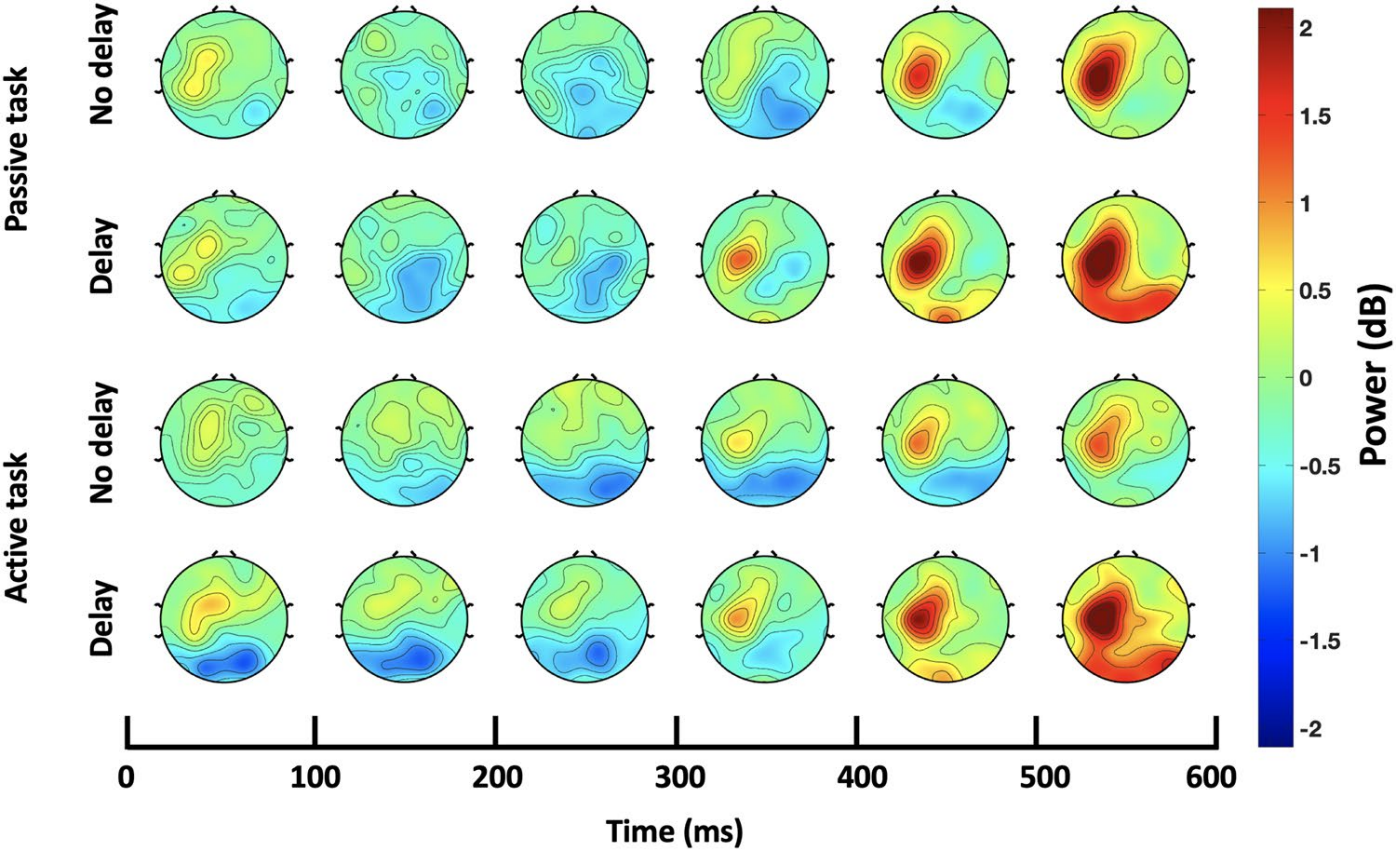
Time course of scalp topographies showing beta power during the passive and active tasks. The scalp topographies under the delayed and synchronous conditions are shown with a resolution of 100 ms. The onset at 0 ms corresponds to the haptic collision event. Beta rebound activation in the sensorimotor ROI is observed time-locked to the haptic collision event in both the passive and active tasks.

**Figure 4:**
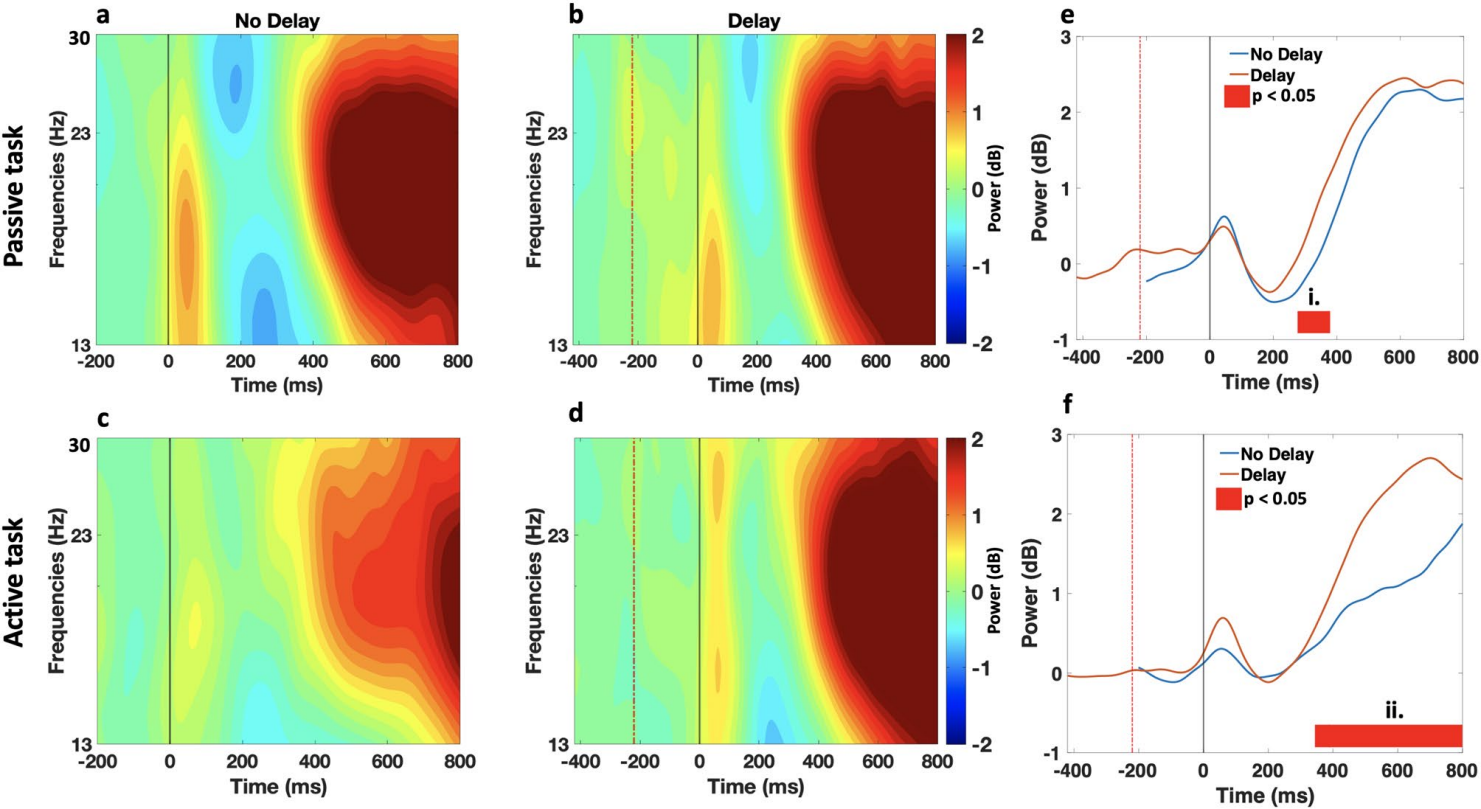
Contralateral sensorimotor ROI time-frequency data. (**a**–**d**) ERSP dynamics of the EEG data in the sensorimotor ROI averaged over trials and subjects per condition and task. Time 0 ms corresponds to the haptic collision event denoted with a grey (solid) line, while the red (dashed) line denotes the event of the visual collision in the delayed condition. A beta rebound is observed under all conditions starting around 200 ms from the haptic collision onset. (**e**, **f**) Time-course of beta power (13–30 Hz) under the no delay (blue) and delay (orange) conditions time-locked to the haptic collision. Both waveforms are plotted starting with their corresponding baseline (200 ms before the visual collision). Significant time window is (i. 280–380 ms; $\textit{p}<$0.05, randomization test, FDR corrected) for the passive task and (ii. 360–800 ms; $\textit{p}<$0.05] , randomization test, FDR corrected) for the active task.

At the contralateral sensorimotor ROI, a post-movement beta rebound time-locked to the haptic collision event was observed. The time course of scalp topographies shown in Fig. 3 clearly depicts the beta rebound under the four conditions (passive/active, with/without haptic delay). Regardless of the condition, an examination of the time-frequency EEG data at the contralateral sensorimotor ROI revealed that beta rebound starts around 200 ms post the haptic collision, as shown in Fig. 4. During the passive task, beta rebound takes almost a similar form under the synchronous and the delayed conditions. Statistical tests confirm this, as the duration at which there is statistically significant difference in the rebound is relatively short ($\textit{p}$ = 0.0284, randomization test, FDR corrected; $t_{range}$ = [280–380 ms]). On the other hand and during the active task, beta rebound differs significantly between the synchronous and delayed conditions for a much longer duration as can be seen in Fig. 4f ($\textit{p}$ = [0.0105–0.0452] , randomization test, FDR corrected; $t_{range}$ = [360–800 ms]). It can be observed that even though the rebound is time-locked to the haptic collision event in the active task, the rate at which the beta rebound occurred in the synchronous condition is slower than that of the delayed condition.

### Cognitive correlates

**Figure 5:**
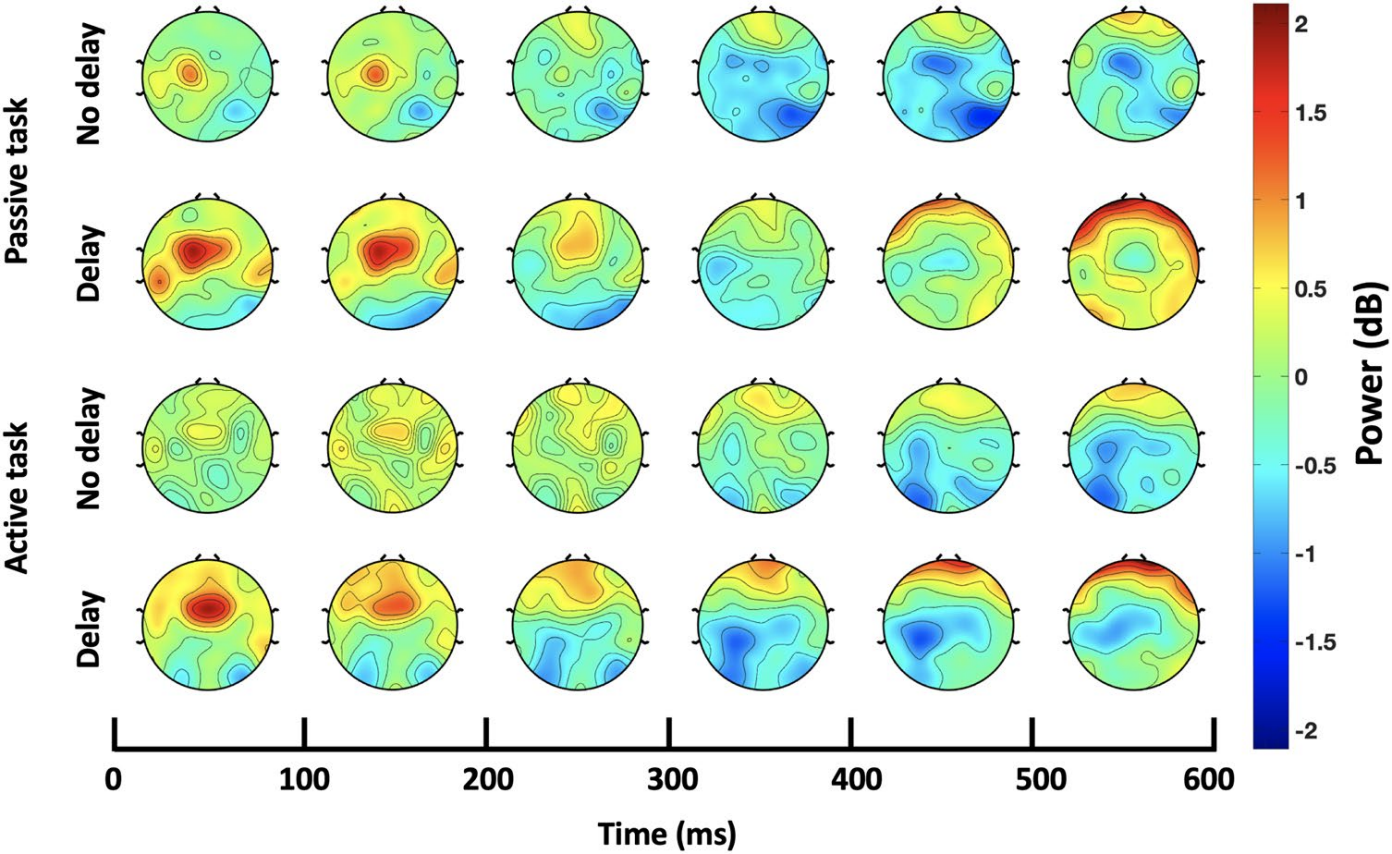
Time course of scalp topographies showing theta power during the passive and active tasks. The scalp topographies under the delayed and synchronous conditions are shown with a resolution of 100 ms. The onset at 0 ms corresponds to the haptic collision event. Theta activation in the midfrontal ROI is observed with a stronger intensity after the introduction of the haptic delay in both, the passive and active tasks.

**Figure 6:**
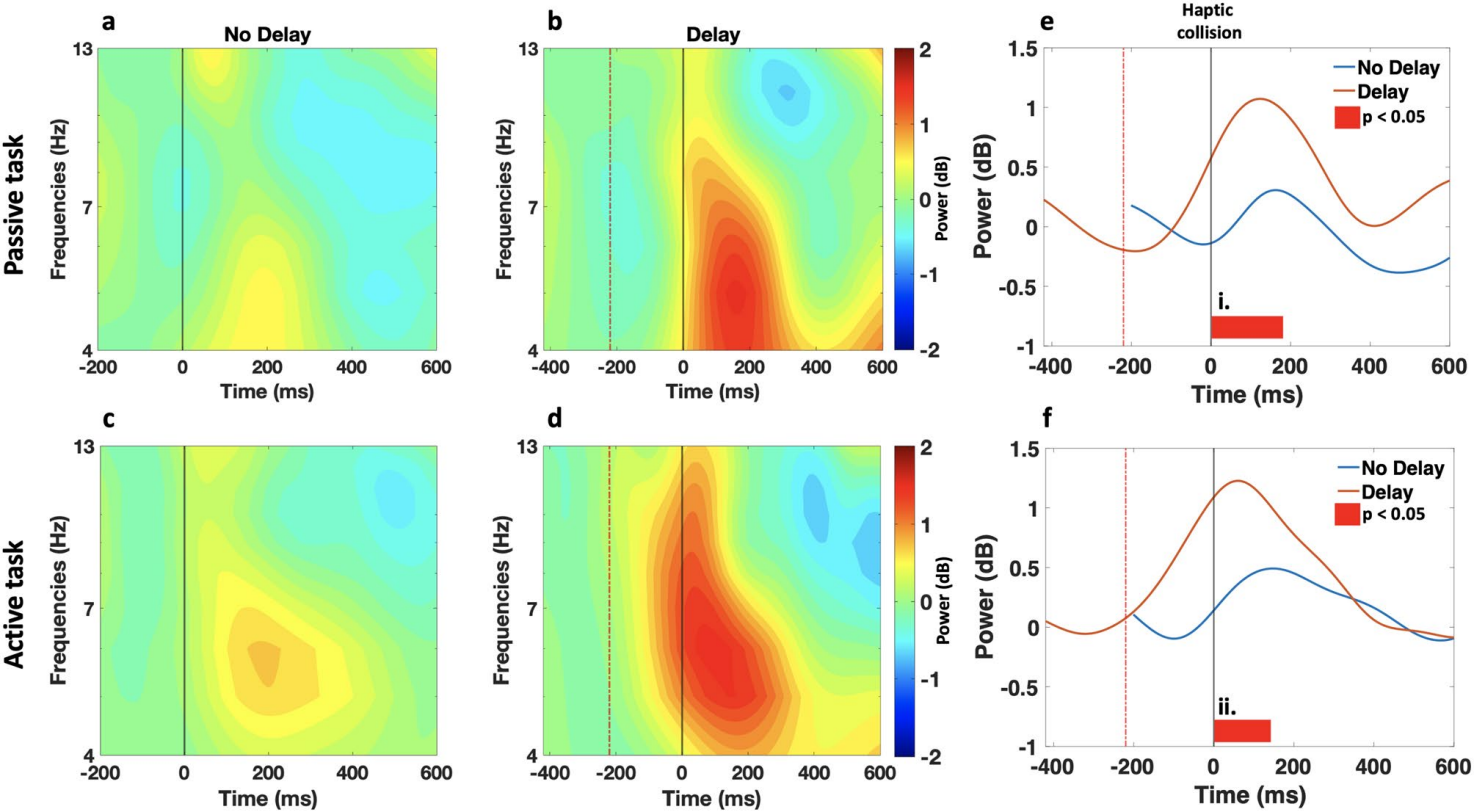
Midfrontal ROI time-frequency data (**a**–**d**) ERSP dynamics of the EEG data in the midfrontal ROI averaged over trials and subjects per condition and task. Time 0 ms corresponds to the haptic collision event denoted with a grey (solid) line, while the red (dashed) line denotes the event of the visual collision in the delayed condition. (**a**,**c**) Theta band activation centered around 200 ms is observed in the synchronous condition. (**b**, **d**) As a result of the haptic delay, theta activation power is increased. Under the delayed condition, theta activation pattern during the passive task differs from that during the active task. Under the delayed condition and during the passive task, theta activation sharply increases around the introduction time of the delayed haptic feedback. In contrast, during the active task, theta activation increases earlier, right after the visual collision and the missed haptic feedback. (**e**, **f**) Time-course of theta power (4–9 Hz) under the no delay (blue) and delay (orange) conditions time-locked to the haptic collision. Both waveforms are plotted starting with their corresponding baseline (200 ms before the visual collision). Significant time window is (i. 0–180 ms; $\textit{p}_{range}<0.05$, randomization test, FDR corrected) for the passive task and (ii. 0–140 ms; $\textit{p}<0.05$, randomization test, FDR corrected) for the active task.

The time course of scalp topographies of theta band showed an evident activation that was modulated by the haptic delay localized and exhibited at the midfrontal ROI as shown in Fig. 5. An examination of the time-frequency EEG data at the midfrontal ROI revealed theta power synchronization in both the passive and active tasks following the haptic collision event, as shown in the ERSPs plots in Fig. 6a–d. From a spectral perspective and in the presence of delay, theta activation in the passive task extends from 4 to 9 Hz, while in the active task, the activation extends from theta to alpha band, reaching $\sim 13$ Hz. Temporally, theta synchronization is delayed with the introduction of haptic delay, indicating an association with the haptic collision event. During the active task and in the presence of delay, theta activation peaks earlier than that in the passive task (passive: t = 125 ms; active: t = 60 ms) with respect to the haptic collision event. More specifically, in the active task, theta activation starts before the delayed haptic feedback occurs. In contrast, in the passive task, theta activation is strengthened almost when the delayed haptic feedback is delivered. In the absence of haptic delay (Fig. 6a,c), an activation centered around 200 ms in the theta band is observed regardless of the task type.

The time course of the mean theta power in the midfrontal ROI is presented in Fig. 6e,f. It can be observed that theta synchronization is significantly higher under the presence of the haptic delay, regardless of the task type (passive: $\textit{p}_{range} =[0.016, 0.037]$, randomization test, FDR corrected; $t_{range}$ = [0–180 ms] and active: *p*=0.021, randomization test, FDR corrected; $t_{range}$ = [0–140 ms]).

**Figure 7:**
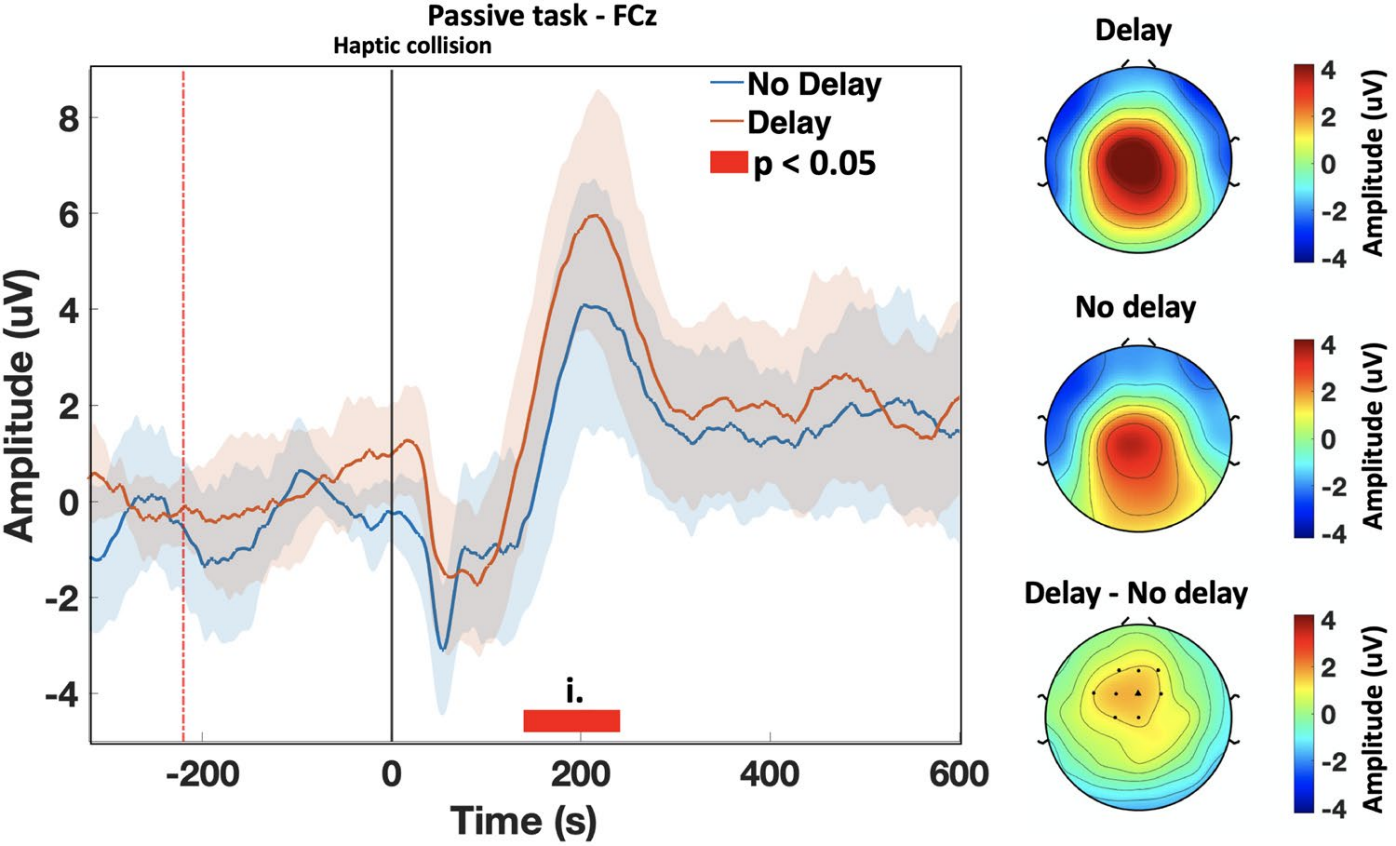
The ERP onset at 0 ms represents the haptic collision event and is denoted by a grey (solid) vertical line. A P200 peak is observed at FCz and surrounding electrodes. The P200 peak is in response to the haptic collision; it is delayed by 220 ms in accordance with the delayed haptic stimulus (orange). A higher mean amplitude peak is observed under the presence of haptic delay compared to the synchronous case with ($p<0.05$, randomization test, FDR corrected; $t_{range}$ = [140–240 ms]). Thick waveforms represent the mean ERP (across trials and participants), while the shaded areas represent the standard deviation for each condition. The topography plot (right panel) for the delayed condition shows a stronger activation around the central cortex when compared to the synchronous condition. The difference topography plot denotes the channels at which a statistically significant difference was observed; FCz is marked as a triangle. All topography plots represent the mean activation between 140 and 240 ms.

The following analysis aims to investigate the ERP activations at the FCz electrode, which is part of the midfrontal ROI as well. At FCz, a sharp ERP peak is observed around 200 ms time-locked to the haptic collision. The P200 peak is delayed under the presence of the haptic delay; Fig. 7 shows the P200 peak at FCz during the passive task, with the onset being the haptic collision event. The P200 peak is commonly observed between 140 and 280 ms^40–42^ post the onset. It was found that the amplitude of the P200 is significantly larger in the presence of the haptic delay during the passive task ($\textit{p}_{range} =[0.011, 0.048]$, randomization test, FDR corrected; $t_{range}$ = [140–240 ms]). Additionally, the mean activation topography of the scalp from 140 to 240 ms is shown in Fig. 7 under the synchronous condition, the delayed condition, and the difference between them. The difference topography plot shows the channels with a P200 amplitude that is significantly different between the synchronous and delayed conditions. A negative peak (N100) precedes the P200 component at around 80 ms after the haptic collision; this peak, however, is not significantly modulated by the presence of the haptic delay. During the active task, no statistically significant difference was found between the P200 peak amplitude of the delayed condition and the synchronous condition.

**Figure 8:**
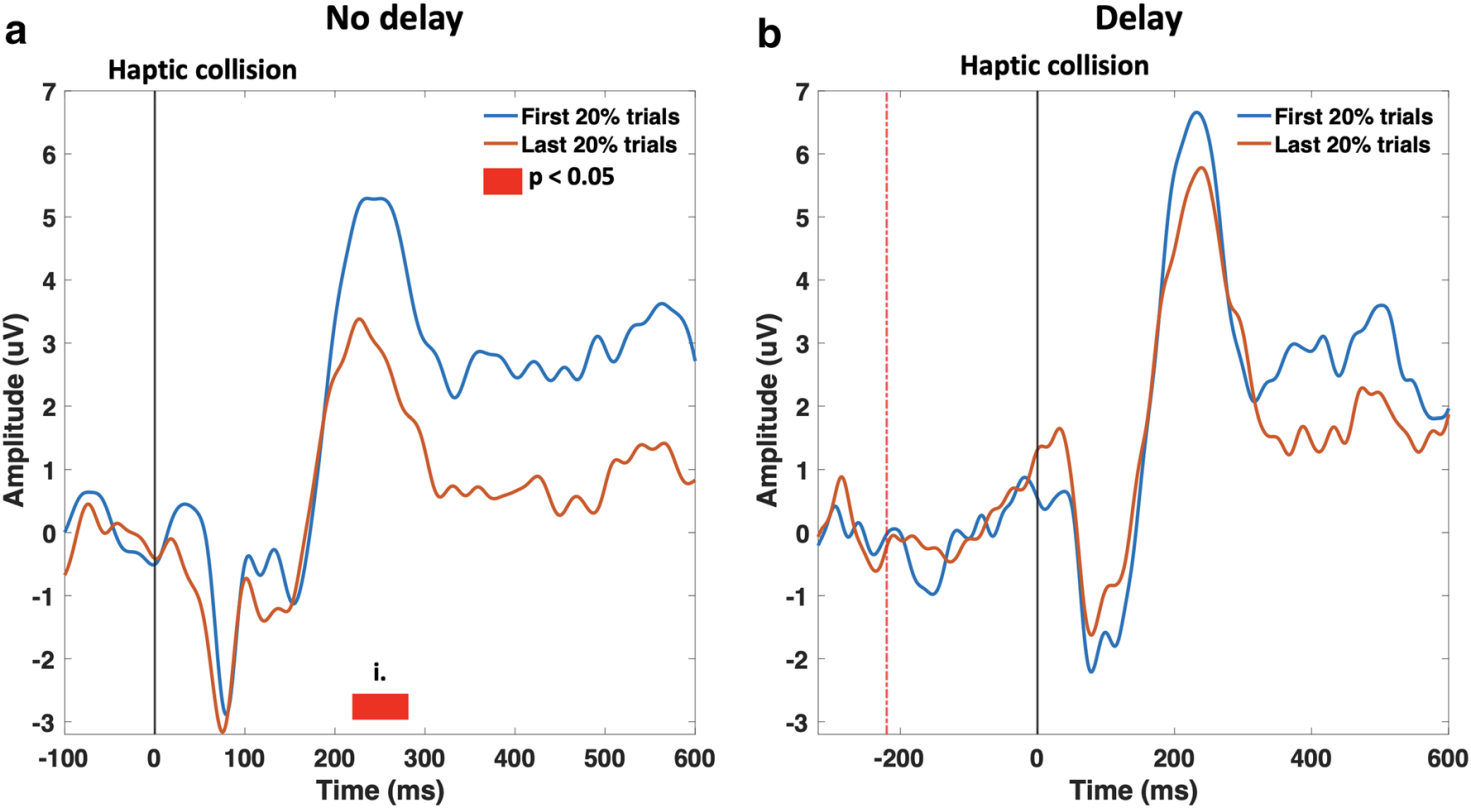
Learning effect due to repetitive exposure to the haptic collision across trials during the passive task. (**a**) P200 peak amplitude significantly ($p<0.05$, randomization test, FDR corrected; $t_{range}$ = [220–280 ms]) reduces in the last ten trials in the absence of haptic delay compared to the first ten trials. (**b**) In the presence of haptic delay, however, there is no statistical significant difference between the first 10 and last 10 trials’ peak amplitude ($p>0.05$, randomization test, FDR corrected; $t_{range}$ = [220–280 ms]).

The learning effect during the passive task and its impact on the P200 component has been analyzed in the synchronous and delayed conditions as shown in Fig. 8. The mean ERP of the first 20% trials of each condition per subject were compared against the mean ERP of the last 20% trials of the same condition per subject; P200 peak modulation has been examined. Figure 8 shows a significant reduction ($\textit{p}_{range} =[0.017, 0.034]$, randomization test, FDR corrected; $t_{range}$ = [220–280 ms]) in the P200 peak amplitude during the passive task under the absence of haptic delay. In the presence of delay, on the contrary, no significant modulation was observed. Hypothesis (3) is therefore accepted ($\textit{p}_{range} =[0.400, 0.791]$, randomization test, FDR corrected; $t_{range}$ = [220–280 ms]) indicating no statistical difference between the first and last 20% of the trials in the presence of haptic delay in the passive task. During the active task, no significant modulation of the P200 peak has been observed over the trials.

### Performance evaluation

Participants were occasionally asked after trials if the haptic collision was delayed or not. This is to keep the participant engaged with the experimental task and avoid boredom which could occur due to the repetitive nature of the EEG experiments. In total, every participant was asked to answer this question 30 times (15 for the passive task and 15 for the active task) during the whole session out of 200 trials. The average percentage of misperceptions is 11.2% for the passive task and 11.5% for the active task, with no significant difference observed in misperception between the two tasks. Cumulatively, participants were accurate in their answers (and consequently their perception of the delay) on average 88.6% of the time (Min 66.6%, Max 100%, Median 93.3%). The majority of the participants (74%) were not familiar with using a haptic device, so the training session was essential. Around 58% of the participants were able to identify better the presence of the haptic delay in the passive task as compared to 42% who were able to identify better the presence of the haptic delay in the active task based on the questionnaire.

## Discussion

Haptic delay is a vital perceptual attribute that can drastically affect the quality of experience in many human–machine interaction scenarios such as in virtual reality and haptic-skill transfer applications^54^. The goal of the present study was to investigate the sensory and cognitive neural correlates of the haptic delay during passive and active haptic tasks. The main finding is that haptic delay induced significantly different neural traces than the synchronous condition in both ERP and oscillatory activity (ERSPs), mainly at the midfrontal region. Processes involved include multisensory divided attention, conflict resolution, and perceptual attention.

With respect to sensory ERSP activity, beta rebound starting time was found to be time-locked to the haptic collision. Post movement beta rebound is generally characterized by a pronounced increase in beta power occurring within 1000 ms post the movement^43^. Beta rebound is reported to be most prominent over the sensorimotor cortical areas^55^ once the movement of the effector (e.g: limb) is terminated^56^. Several studies also showed that beta rebound is not only observed during active movements, it is also clearly exhibited during passive^57–59^ or imagined movements^60,61^. Indeed, in this study, we observed beta rebound under both conditions, the passive and active task. The functional role of beta rebound is not fully agreed upon in the literature and is still under debate; however, a widely accepted hypothesis is that it inhibits the motor network upon the movement termination^62,63^. The current findings show in the passive task, beta rebound is relatively similar between the synchronous and delayed conditions. Participants receive the haptic feedback while their hands are at rest with merely a 220 ms delay in time under the delayed condition. It is thus expected from a sensory perspective, both scenarios are equivalent, and beta rebound is not modulated. The active task is intrinsically different as the haptic feedback is delivered during the hand movement. Under the delay condition, the wrist stops moving around the time of the visual collision indicating a successful end to the trial from the participant’s perspective. Thus, the haptic feedback is delivered when the wrist is relatively stationary, mimicking the passive condition from a sensory perspective. However, in the synchronous case, the haptic collision is felt while the wrist is still moving and about to stop. Since the direction of the hand movement and the direction of the haptic collision are opposite, we believe that the inhibitory processes will occur at a decreased rate compared to the other three conditions, as shown in Fig. 4f.

In the midfrontal ROI, theta oscillation was found to peak in a relatively similar manner during the passive and active tasks in the absence of haptic delay as shown in Fig. 6e,f. When more than one modality is in use, theta oscillations are considered a marker of multisensory divided attention^17,19^. Previous studies reported a relationship between theta oscillations and various functions related to multisensory divided attention in audio–visual integration^19,64^. In the context of our study, haptic-visual modalities are both employed, which is a different form of multisensory activity from the studies mentioned above^19,64^. Despite the difference in the modalities involved, theta oscillation was found to increase after the onset of the haptic-visual event (synchronous condition). Introducing haptic delay to the ball-racket collision event caused a significant increase in the theta oscillation in the midfrontal cortex and an observed delay of the peak power as clearly shown in Fig. 6b,d. In this scenario, divided attention is still required due to the dual nature of the stimuli (visual-haptic); however, further cognitive processes are induced due to the incongruity between the two modalities. Indeed, theta oscillation was reported to be more pronounced under incongruent cross-modal stimulation as compared to congruent stimulation under visuotactile^24,25^ and audiovisual stimulations^21^.

These studies^21,24,25^ suggest that theta oscillation reflects conflict processing and resolution since only in the incongruent condition, a bimodal competition of stimuli was present. In our current study, participants typically would expect the haptic feedback to be delivered and felt at the moment of the visual collision; an absence of this anticipated sensation at the moment of collision followed by an unexpected haptic stimulation once the collision is over would initiate neural processes of conflict detection and resolution. In other words, it seems that theta band activation in the middle frontal cortex index perceived multisensory incongruence and induced conflict detection mechanisms accordingly. In this light, it is tempting to state that conflict processing resembled in theta oscillation was possible to observe due to the recognizable delay by the human brain (220 ms).

In the presence of delay and comparing theta oscillation during the passive and active tasks, it is observed from Fig. 6b,d that theta oscillation peaks earlier in the active task. Understanding the differences between the active and passive movements proves helpful in understanding the underlying neural differences during the two tasks. A major difference between the two lies in the mechanisms involved in proprioception, a sense of awareness of the spatial and mechanical status of the body^65^. During the passive task, a one-fold dominance of proprioception is in action (awareness of body position), while during the active task, two-fold proprioception is in action (awareness of body position and movement). We argue that the delay in theta oscillation is caused by the delay in the realization process of the conflict. This realization occurred earlier in the active task as compared to the passive task due to the additional mechanism in action in the active task under proprioception. The two-fold continuous proprioceptive feedback activated during the active task could have caused an earlier realization of the incongruity conflict between the two modalities. As argued in self-reporting studies, it is observed that the detection threshold of the haptic delay with respect to the corresponding visual stimulus is smaller during active tasks when compared to passive tasks^6^.

From a spectral perspective, it can be observed from Fig. 6b,d that theta activation is limited between 4 and 9 Hz during the passive task, while in the active task, the activation extends beyond the theta band to cover the alpha band as well (4–13 Hz). It is interesting to observe that this activation in the alpha band is not a separate form of activation; instead, the activation looks like a single body covering both theta and alpha band. A similar observation is found in another work that studied error processing, and stimuli congruence^66^ where participants were asked to prioritize accuracy in detecting incongruence. It is argued that alpha activation in the frontal region associates with top-down attentional control over lower sensory regions as a reorienting response^67^. When sustained attention is required, participants need to keep track of their attention, and in case of any lapses, refocus. This refocusing process is attributed to the observed alpha synchronization. In light of this study, and to explain the alpha activation in Fig. 6d, we speculate that there is a relationship between the nature of the active task and the need for higher attentional control over the sensory channels. Indeed, due to the motor movement involved during the active task, higher attentional control could be needed to detect the conflict caused by the haptic delay.

In the time domain, attention-related ERP component (P200 peak) was observed particularly at the midfrontal ROI. P200 peak is observed to be time-locked to the haptic stimulus; a delay in the haptic feedback caused the same delay to the P200 peak. A study that focused on studying passive ankle movements found that the P200 peak is a later component that is related to endogenous factors and could reflect cognitive processes such as attention and haptic stimulus localization or estimation^68^. In fact, P200 is reported to be an ERP component that is highly related to early attentional mechanisms^69,70^. In this study, a significant P200 modulation ($p<0.05$, randomization test, FDR corrected) was observed under the presence of haptic delay in the passive task only. This suggests that haptic delay had a significant effect on attention towards the haptic feedback during the passive task, while the same is not true during the active task. A possible explanation is that participants are perceptually less occupied during the passive task, which makes them more sensitive and attentive towards the haptic feedback when delayed. The impact of the learning effect on attention and high order perception (i.e.: P200 amplitude) was examined. As mentioned earlier in the results section, P200 amplitude was significantly reduced in the last 20% trials during the passive task and in the absence of haptic delay. Participants exhibited a strong learning effect under the passive condition in the absence of the haptic delay, and their attention (P200 peak) is reduced. Conversely, participants did not exhibit a learning effect in the presence of haptic delay (passive task) and elicited almost the same attention at the start and towards the end of the trial set. During the active task, participants were less susceptible to the learning effect in the presence or absence of the haptic delay.

One limitation of this study is that a single haptic delay value (220 ms) was tested. However, it is crucial to start with an easily recognizable delay that will highlight the related neural correlates of delay, which can assist in understanding the neural traces for granulated values of delay. Future studies should build on the obtained results and understand the effect of haptic delay on a continuous scale. Additionally, jitter is another parameter that is quite important in altering the haptic experience and is a potential future direction. In sum, the present study provides evidence that haptic delay induces significant neural traces that could be observed over the central and the midfrontal ROI during active and passive tasks. More work is warranted to cover other perceptual attributes important during haptic communication.

## Data Availability

The EEG dataset analysed and discussed in this work can be obtained from the corresponding author upon reasonable request.
